# Concurrent intra-aortic balloon pump and veno-arterial extracorporeal membrane oxygenation for acute coronary syndrome-related cardiogenic shock: A meta-analysis of multivariate studies

**DOI:** 10.17305/bb.2024.11011

**Published:** 2024-10-18

**Authors:** Xin Huang, Di Huang, Weiye Wan, Hongling Zhang, Zhengdong Liu

**Affiliations:** 1 Department of Critical Care Medicine, Affiliated Lu’an Hospital, Anhui Medical University, Lu’an, China; 2 The First Clinical Medical College, Lanzhou University, Lanzhou City, China; 3 Wannan Medical College, Wuhu, China; 4 Department of Critical Care Medicine, Union Hospital, Tongji Medical College, Huazhong University of Science and Technology, Wuhan, China

**Keywords:** Cardiogenic shock, acute coronary syndrome, extracorporeal membrane oxygenation, intra-aortic balloon pump, mortality

## Abstract

Concurrent intra-aortic balloon pump (IABP) use has been suggested to reduce mortality in patients with acute coronary syndrome (ACS)-related cardiogenic shock (CS) on veno-arterial extracorporeal membrane oxygenation (ECMO). However, this observation is primarily based on small-scale univariate studies. The aim of this meta-analysis was to evaluate whether concurrent IABP and ECMO were independently associated with reduced mortality in patients with ACS-related CS. We searched Medline, Web of Science, and Embase for studies published up to May 28, 2024. The inclusion criteria were longitudinal observational studies comparing concurrent IABP and ECMO to ECMO alone in ACS-related CS patients, reporting all-cause mortality with multivariate adjustments. The primary outcome was the risk ratio (RR) of short-term mortality. A random-effects model incorporating heterogeneity was used to pool the results. Seven cohort studies, involving 5467 patients, were included. Concurrent IABP and ECMO were associated with a significant reduction in short-term mortality (adjusted RR: 0.64; 95% CI: 0.48–0.87, *P* ═ 0.005; *I*^2^ ═ 83%). Sensitivity analyses confirmed the robustness of these results. Meta-regression indicated that the proportion of men in each study significantly influenced the outcomes, fully explaining the heterogeneity (*I*^2^ residual ═ 0%). Subgroup analyses showed consistent results across various study designs, patient ages, observational durations, and study quality scores. In conclusion, concurrent IABP and ECMO are independently associated with reduced short-term mortality in ACS-related CS patients, particularly in studies with higher proportions of men. These findings support the potential benefits of combined mechanical support in this high-risk population.

## Introduction

Acute coronary syndrome (ACS), particularly acute myocardial infarction (AMI), can lead to cardiogenic shock (CS), a life-threatening condition where the heart is unable to pump enough blood to meet the body’s needs, resulting in multi-organ failure and high mortality rates [1, 2]. Despite advances in medical therapies and revascularization techniques [3], mortality rates for ACS-related CS remain alarmingly high [4, 5]. Veno-arterial extracorporeal membrane oxygenation (VA-ECMO) has emerged as a key intervention in managing severe CS, providing temporary circulatory and respiratory support, and allowing time for myocardial recovery or bridging to more definitive treatments [6, 7]. However, while extracorporeal membrane oxygenation (ECMO) provides significant hemodynamic support, short-term mortality for patients with ACS-related CS remains high, and the treatment strategies for these patients need further optimization [8]. The intra-aortic balloon pump (IABP) is a mechanical support device that inflates and deflates in the aorta, enhancing coronary perfusion and reducing left ventricular afterload [9, 10]. In ACS-related CS, particularly in patients receiving VA-ECMO, IABP may provide additional hemodynamic benefits [11]. By improving myocardial oxygen supply and decreasing cardiac workload, IABP could potentially enhance cardiac function and improve patient outcomes [12]. Previous pilot studies and meta-analyses [13–15] have suggested that the combined use of IABP and ECMO may reduce mortality in this patient population. To our knowledge, no randomized controlled trials (RCTs) have been conducted to assess the role of combined IABP and ECMO use in patients with ACS-related CS. Furthermore, prior meta-analyses on this topic have primarily used crude mortality data [14, 15], which may not fully account for the complex interplay of risk factors affecting patient outcomes. For example, the recent meta-analysis by Liu et al. [15] made a valuable contribution to the field but focused mainly on crude data. In our study, we performed a meta-analysis that exclusively included studies with multivariate adjustments to address this limitation, controlling for key confounders, such as age, sex, ACS subtype, and renal function. This methodological refinement is essential for providing a more accurate assessment of the efficacy of combined IABP and VA-ECMO on mortality outcomes in ACS-CS patients. This approach offers new insights and contributes to a more nuanced understanding of the factors influencing mortality in ACS-CS patients.

## Materials and methods

We followed the PRISMA 2020 guidelines [16, 17] and the Cochrane Handbook for Systematic Reviews and Meta-Analyses [18] throughout the study, including study design, data collection, statistical analysis, and interpretation of results. This meta-analysis was registered on the Open Science Framework under the registration identifier osf.io/c27fz.

### Literature search

To identify studies relevant to our meta-analysis, we searched Medline, Web of Science, and Embase using comprehensive search terms, including (1) “extracorporeal membrane oxygenation” OR “ECMO” OR “heart-lung machine” OR “extracorporeal life support” OR “ECLS”; (2) “intra-aortic balloon pump” OR “Intraaortic balloon pump” OR “IABP” OR “counterpulsation device”; (3) “shock” OR “cardiogenic shock” OR “cardiac shock”; (4) “myocardial infarction” OR “acute coronary syndrome” OR “ACS” OR “AMI”; and (5) “combined” OR “concomitant” OR “concurrent” OR “simultaneous” OR “simultaneously” OR “controlled” OR “matched” OR “adjusted” OR “adjustment” OR “adjusting” OR “logistic” OR “multivariate” OR “multivariable” OR “confounding” OR “propensity”. The search was limited to studies involving human subjects. Detailed search strategies for each database are available in Supplemental Material 1. Only studies published as full-length articles in peer-reviewed journals in English or Chinese were included. Additionally, we manually reviewed references from relevant original and review articles to identify other potential studies. The search included literature published up to May 28, 2024.

### Inclusion and exclusion criteria

Eligible studies had to meet the following criteria: (1) observational or longitudinal follow-up studies, including prospective and retrospective cohort studies, nested case-control studies, and post-hoc analyses of clinical trials; (2) studies involving patients with ACS-related CS treated with VA-ECMO; (3) evaluation of concurrent treatment with IABP and VA-ECMO; (4) studies comparing patients treated with ECMO alone; (5) studies reporting short-term all-cause mortality during hospitalization or within a follow-up period of up to three months; and (6) studies that compared the relative risk of all-cause mortality between patients with and without concurrent IABP, based on ECMO treatment, with at least age and sex adjustments in a multivariate analysis. Exclusion criteria were: (1) cross-sectional studies; (2) studies of patients with CS not related to ACS; (3) studies without VA-ECMO patients; (4) studies that did not evaluate the effects of concurrent IABP and ECMO; (5) studies that did not compare outcomes to ECMO-only patients; (6) studies that did not report all-cause mortality outcomes; (7) studies with univariate outcome data only; and (8) studies published as conference abstracts, unpublished data, reviews, or editorials. If studies had overlapping populations, we included the one with the largest sample size for the meta-analysis.

### Study quality evaluation and data extraction

The literature search, study identification, quality assessment, and data collection were performed independently by two authors. In case of disagreement, the corresponding author was consulted to resolve the issue. We evaluated the quality of the included studies using the Newcastle–Ottawa Scale (NOS) [19], which assesses three key aspects: selection of the population, control of confounders, and outcome measurement/analysis. NOS scores ranged from 1 to 9, with 9 indicating superior quality. Data extracted from each study included: study information (author, year, country, and design), patient characteristics (diagnosis, sample size, age, sex, and proportion receiving percutaneous coronary intervention [PCI]), number of patients with concurrent IABP and ECMO, number of patients with ECMO only, length of observation, number of deaths, and variables matched or adjusted for in multivariate analyses.

### Ethical statement

Ethical approval was not required for this study according to local/national guidelines. Written informed consent to participate was also not required under these guidelines.

### Statistical analysis

The influence of concurrent IABP on the mortality risk of patients with ACS-related CS treated with ECMO was summarized as a risk ratio (RR) with corresponding 95% confidence intervals (CIs). For studies reporting odds ratios (ORs), the data were converted to RRs for the meta-analysis using the formula: RR ═ OR / ([1 − pRef] + [pRef × OR]), where *pRef* is the incidence of all-cause mortality in the reference group (ECMO only group) [20]. RRs and their standard errors were then computed from the 95% CIs or *P* values, followed by logarithmic transformation for variance stabilization. Heterogeneity among studies was assessed using the Cochrane *Q* test and *I*^2^ statistics [21], where an *I*^2^ > 50% indicated significant heterogeneity. The findings were combined using a random-effects model to account for the influence of heterogeneity [18]. Sensitivity analyses, excluding one dataset at a time, were conducted to assess the robustness of the results. Univariate meta-regression analyses were performed to evaluate the influence of study characteristics (as continuous variables) on the outcome, such as sample size, mean age, proportion of men, proportion of patients with PCI, and study quality (as evaluated by NOS). Predefined subgroup analyses examined how study characteristics influenced outcomes, using median values of continuous variables to define subgroups. Publication bias in the meta-analysis was assessed by constructing funnel plots and visually inspecting their symmetry [22]; Egger’s regression test was also performed [22]. Statistical analyses were conducted using RevMan (version 5.1; Cochrane Collaboration, Oxford, U.K.) and Stata software (version 12.0; Stata Corporation, College Station, TX, USA).

## Results

### Study inclusion

**Figure 1.**
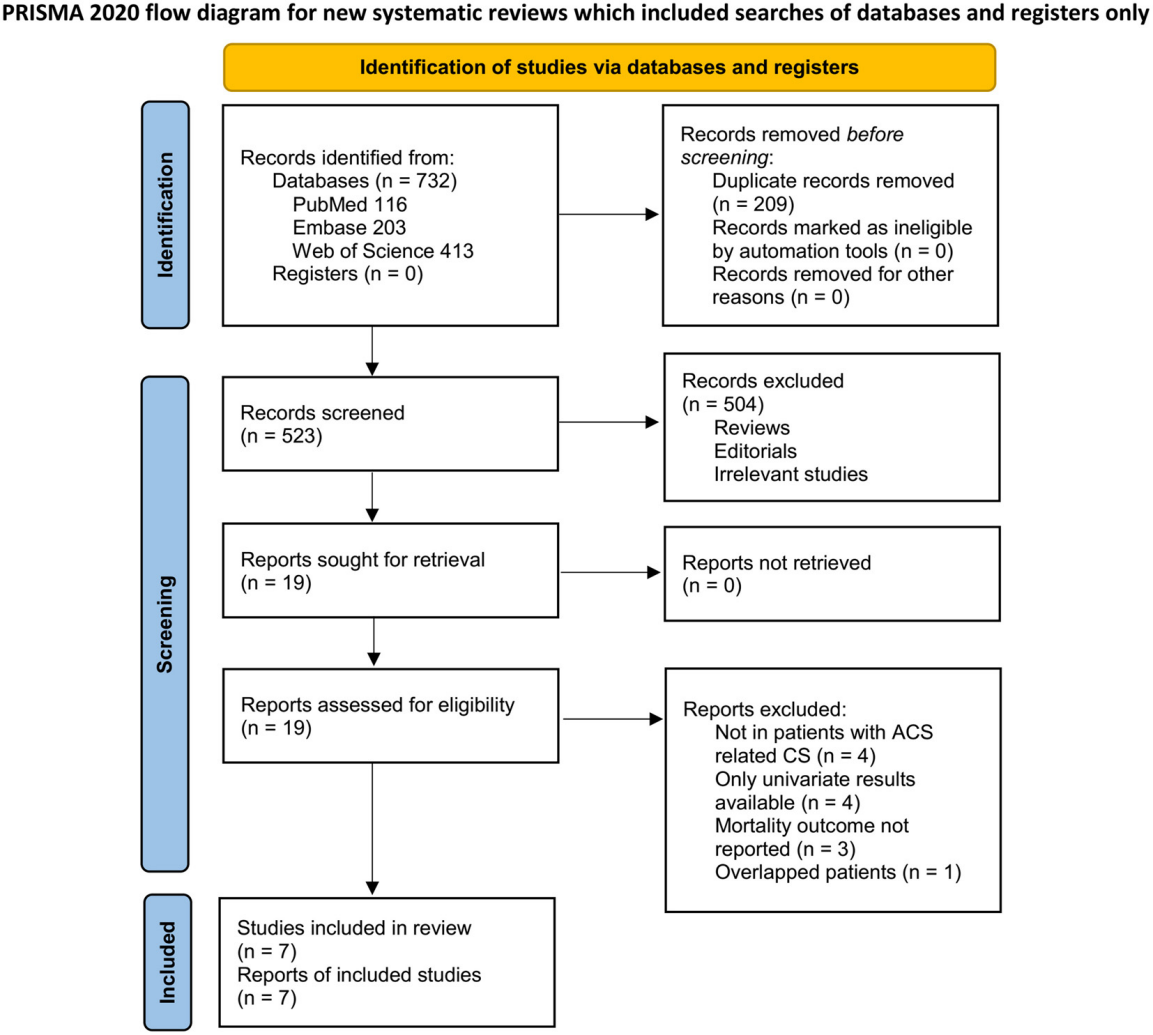
The PRISMA flow diagram of database search and study inclusion.

The process of study inclusion is shown in Figure 1. Initially, 732 potentially relevant records were identified through comprehensive searches of three databases, and 209 were excluded due to duplication. Screening of titles and abstracts for the remaining records excluded 504 studies, mostly because they were not relevant to the meta-analysis. The full texts of the 19 remaining studies were then reviewed by two independent authors, with 12 excluded for reasons listed in Figure 1. Seven cohort studies were deemed suitable for quantitative analyses [23–29].

### Overview of study characteristics

Table 1 summarizes the characteristics of the included studies. Overall, seven cohort studies—five retrospective [23–26, 28] and two prospective [27, 29]—were included. These studies, published between 2014 and 2023, were conducted in Korea, Taiwan, France, Japan, and China. Two studies focused on patients with ACS-related CS [24, 26], while the other five involved patients with AMI-related CS [23, 25, 27–29]. In total, 5467 patients were included, with a mean age ranging from 52.7 to 66.9 years, and the proportion of men ranging from 75.4% to 84.8%. Among these patients, 4292 received concurrent IABP and VA-ECMO, while 1175 received VA-ECMO alone. Short-term mortality outcomes—such as 14-day [24], 30-day [25–27, 29], and in-hospital mortality [23, 28]—were reported across the studies. Multivariable analyses adjusting for age, sex, cardiovascular risk factors, and comorbidities were used to assess the effect of concurrent IABP and ECMO on short-term mortality. The NOS scores for these studies ranged from 7 to 9, suggesting overall good study quality (Table 2).

**Table 1:** Characteristics of the included studies

**Study**	**Study design**	**Country**	**Patient diagnosis**	**Sample size**	**Mean age (years)**	**Men (%)**	**PCI (%)**	**Number of patients with concurrent IABP and ECMO**	**Number of patients with ECMO only**	**Outcomes reported**	**No. of patients died**	**Variables matched or adjusted**
Park, 2014	RC	Korea	AMI associated CS	96	64.8	77.1	81.3	41	55	In-hospital mortality	51	Age, sex, CPR, STEMI, HR, LVEF, and treatment strategies
Lin, 2016	RC	Taiwan	ACS associated CS	236	55.1	75.4	NR	178	58	14-day mortality	NR	Age, sex, BMI, current smoker, pre-existing comorbidity, CVD, severity index, pre-ECMO CPR, peri-ECMO period procedure, and ECMO set-up site
Overtchouk, 2018	RC	France	AMI associated CS	106	52.7	84	74.5	63	43	30-day mortality	67	Age, sex, smoking status, initial recorded rhythm, CA >30 min, SOFA, primary PCI, serum LA, peak troponin, and year of enrolment
Kuroki, 2021	RC	Japan	ACS associated CA	627	64.8	84.8	86.6	538	89	30-day mortality	418	Age, sex, out-of-hospital CA, initial recorded rhythm, time of registry, and location of the coronary culprit lesion
Nishi, 2022	RC	Japan	AMI associated CS	3815	66.9	83.3	100	2964	851	In-hospital mortality	1838	Age, sex, full score Barthel index at admission, Killip classification, the comorbidities, and CA on admission
Kida, 2022	PC	Japan	AMI associated CS	519	66.9	78.5	88.3	459	60	30-day mortality	290	Age, sex, BMI, STEMI, Killip ≥ 2, multi-vessel disease, left main trunk as the infarct-related vessel, AF, malignant arrhythmia, final TIMI grade 3, PPCI, mechanical complication, and timing of entry to the registry
Pan, 2023	PC	China	AMI associated CS	68	59.8	82.4	100	49	19	30-day mortality	29	Age, sex, serum LA, eGFR, and NT-proBNP

PCI: Percutaneous coronary intervention; IABP: Intra-aortic balloon pump; ECMO: Extracorporeal membrane oxygenation; RC: Retrospective cohort; PC: Prospective cohort; AMI: Acute myocardial infarction; CS: Cardiogenic shock; ACS: Acute coronary syndrome; CPR: Cardiopulmonary resuscitation; STEMI: ST-segment elevation myocardial infarction; HR: Heart rate; LVEF: Left ventricular ejection fraction; NR: Not reported; CA: Cardiac arrest; BMI: Body mass index; SOFA: Sepsis-related organ failure assessment; LA: Lactate acid; AF: Atrial fibrillation; TIMI: Thrombolysis in myocardial infarction; eGFR: Estimated glomerular filtration rate; NT-proBNP: N-terminal-pro brain natriuretic peptide.

**Table 2:** Study quality evaluation via the Newcastle–Ottawa Scale

**Study**	**Representativeness of the exposed cohort**	**Selection of the non-exposed cohort**	**Ascertainment of exposure**	**Outcome not present at baseline**	**Control for age and sex**	**Control for other confounding factors**	**Assessment of outcome**	**Enough long follow-up duration**	**Adequacy of follow-up of cohorts**	**Total**
Park, 2014	1	1	1	1	1	1	1	1	1	9
Lin, 2016	0	1	1	1	1	1	1	0	1	7
Overtchouk, 2018	1	1	1	1	1	1	1	1	1	9
Kuroki, 2021	1	1	1	1	1	1	1	1	1	9
Nishi, 2022	0	1	1	1	1	1	1	1	1	8
Kida, 2022	1	1	1	1	1	1	1	1	1	9
Pan, 2023	1	1	1	1	1	1	1	1	1	9

**Figure 2.**
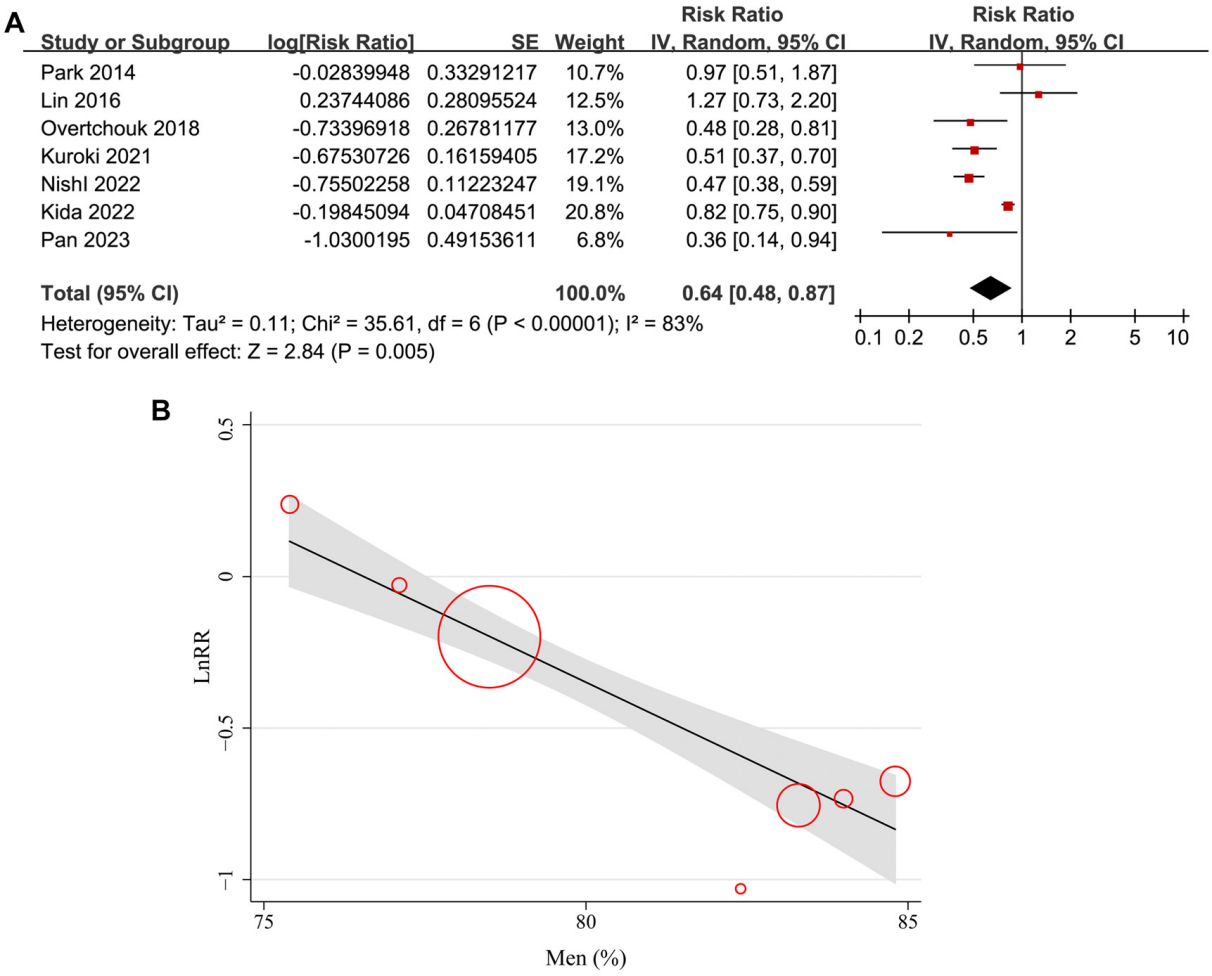
**Forest plots for meta-analysis and meta-regression elucidating the influence of the proportion of men on meta-analysis results.** (A) Forest plots for the overall meta-analysis evaluating the influence of concurrent IABP on short-term mortality of ACS-related CS patients on ECMO; (B) Plots indicating the correlation between the proportion of men in each study on the outcome of the meta-analysis. IABP: Intra-aortic balloon pump; ACS: Acute coronary syndrome; CS: Cardiogenic shock; ECMO: Extracorporeal membrane oxygenation; SE: Standard error; CI: Confidence interval; IV: Inverse variance; LnRR: Log transform of risk ratio.

### Meta-analysis and sensitivity analysis results

Pooled results from the seven cohort studies [23–29] showed that concurrent IABP and ECMO were independently associated with reduced short-term mortality in patients with ACS-related CS (adjusted RR: 0.64, 95% CI: 0.48–0.87, *P* ═ 0.005; *I*^2^ ═ 83%; Figure 2A) compared to patients on ECMO without IABP. Sensitivity analyses—excluding one study at a time—yielded similar results (RR: 0.59–0.70, all *P* < 0.05; Table 3).

### Meta-regression and subgroup analyses results

**Figure 3.**
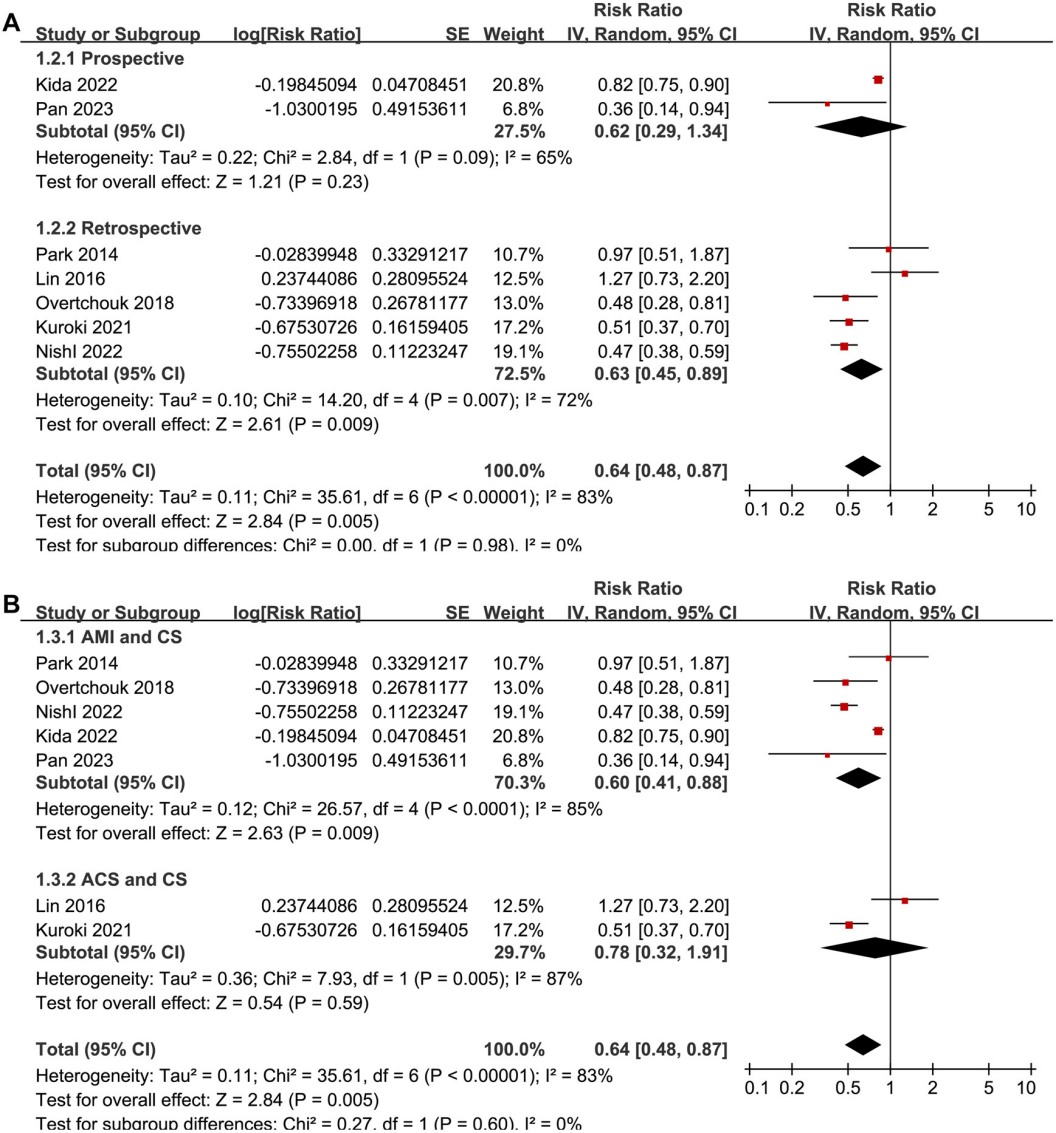
**Forest plots for the subgroup analyses of the influence of concurrent IABP on short-term mortality of ACS-related CS patients on ECMO.** (A) Subgroup analysis according to study design; (B) Subgroup analysis according to the diagnosis of the patients. IABP: Intra-aortic balloon pump; ACS: Acute coronary syndrome; CS: Cardiogenic shock; ECMO: Extracorporeal membrane oxygenation; SE: Standard error; CI: Confidence interval; IV: Inverse variance.

**Figure 4.**
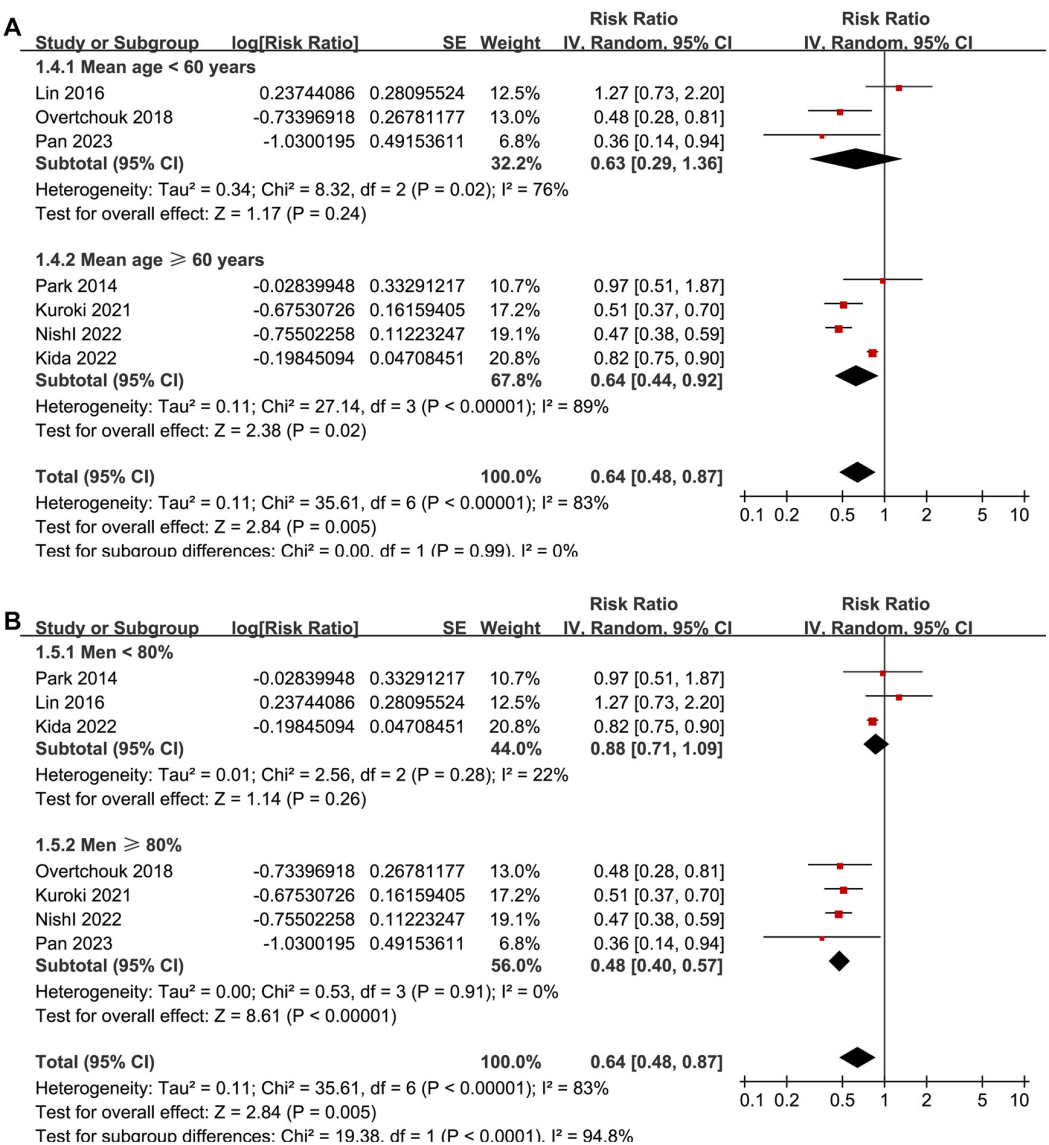
**Forest plots for the subgroup analyses of the influence of concurrent IABP on short-term mortality of ACS-related CS patients on ECMO.** (A) Subgroup analysis according to the mean age of the patients; (B) Subgroup analysis according to the proportion of men in each study. IABP: Intra-aortic balloon pump; ACS: Acute coronary syndrome; CS: Cardiogenic shock; ECMO: Extracorporeal membrane oxygenation; SE: Standard error; CI: Confidence interval; IV: Inverse variance.

**Figure 5.**
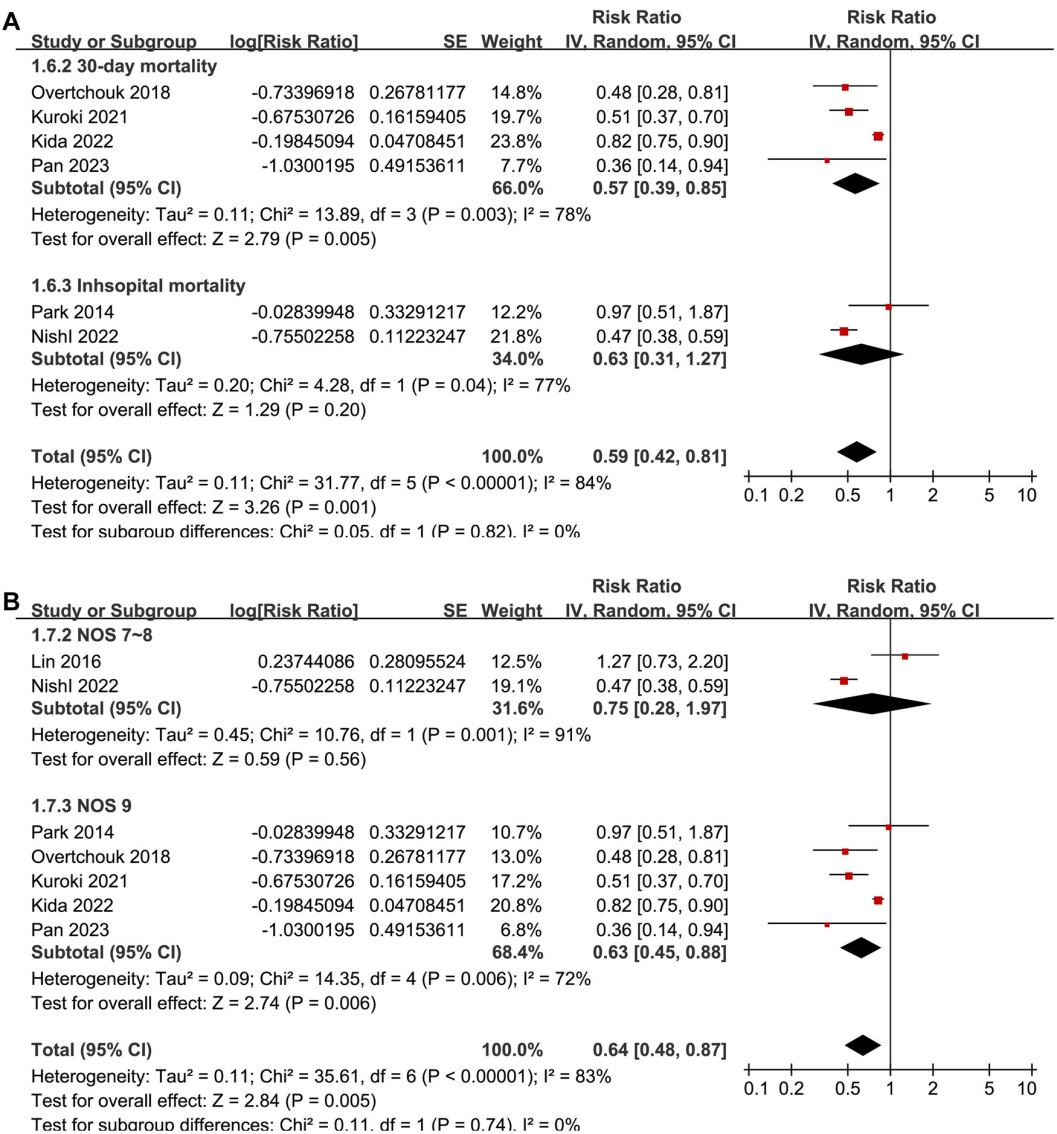
**Forest plots for the subgroup analyses of the influence of concurrent IABP on short-term mortality of ACS-related CS patients on ECMO.** (A) Subgroup analysis according to the observation periods; (B) Subgroup analysis according to the study quality score of each study. IABP: Intra-aortic balloon pump; ACS: Acute coronary syndrome; CS: Cardiogenic shock; ECMO: Extracorporeal membrane oxygenation; SE: Standard error; CI: Confidence interval; IV: Inverse variance.

Univariate meta-regression analyses suggested that the proportion of men in each study was positively correlated with the benefit of concurrent IABP and ECMO in reducing short-term mortality for ACS-related CS patients (coefficient: −0.10, *P* ═ 0.002; Figure 2B and Table 4), explaining the source of heterogeneity (*I*^2^ residual ═ 0%). Other study characteristics, such as sample size, mean age, proportion of PCI patients, or study quality scores, did not significantly affect the results (all *P* > 0.05; Table 4). Subgroup analyses showed similar results between prospective and retrospective cohort studies (RR: 0.62 vs 0.63, *P* for subgroup difference ═ 0.98; Figure 3A), between AMI-related CS and ACS-related CS patients (RR: 0.60 vs 0.78, *P* for subgroup difference ═ 0.60; Figure 3B), and between patients younger or older than 60 (RR: 0.63 vs 0.64, *P* for subgroup difference ═ 0.99; Figure 4A). Interestingly, studies with ≥ 80% male participants showed a more significant benefit of concurrent IABP and ECMO on mortality reduction compared to studies with <80% male participants (RR: 0.48 vs 0.88, *P* for subgroup difference < 0.001; Figure 4B). Finally, subgroup analyses showed similar results for 30-day and in-hospital mortality, and across different study quality scores (*P* for subgroup difference ═ 0.82 and 0.74; Figure 5A and 5B).

**Table 3:** Results of the sensitivity analysis

**Study excluded**	**RR (95% CI)**	***P* for effect**	***I* ^2^**	***P* for heterogeneity**
Park, 2014	0.61 [0.44, 0.85]	0.004	86%	<0.001
Lin, 2016	0.59 [0.42, 0.81]	0.001	84%	<0.001
Overtchouk, 2018	0.67 [0.48, 0.94]	0.02	85%	<0.001
Kuroki, 2021	0.68 [0.48, 0.96]	0.03	83%	<0.001
Nashi, 2022	0.70 [0.51, 0.95]	0.02	71%	<0.001
Kida, 2022	0.60 [0.44, 0.83]	0.002	66%	0.01
Pan, 2023	0.67 [0.49, 0.92]	0.01	85%	<0.001

RR: Risk ratio; CI: Confidence interval.

**Table 4:** Results of meta-regression analysis comparing the influence between concurrent IABP and ECMO vs ECMO alone on short-term mortality in patients with ACS-related CS

**RR for short-term mortality**			
**Variables**	**Coefficient**	**95% CI**	***P* values**
Sample size	−0.00011	−0.00040 to 0.00018	0.37
Mean age (years)	−0.0059	−0.0893 to 0.0775	0.86
Men (%)	−0.10	−0.15 to −0.05	0.002
PCI (%)	−0.017	−0.068 to 0.033	0.42
NOS	−0.23	−0.82 to 0.37	0.37

IABP: Intra-aortic balloon pump; ECMO: Extracorporeal membrane oxygenation; CS: Cardiogenic shock; ACS: Acute coronary syndrome; PCI: Percutaneous coronary intervention; NOS: Newcastle–Ottawa Scale; RR: Risk ratio; CI: Confidence interval.

### Publication bias

**Figure 6.**
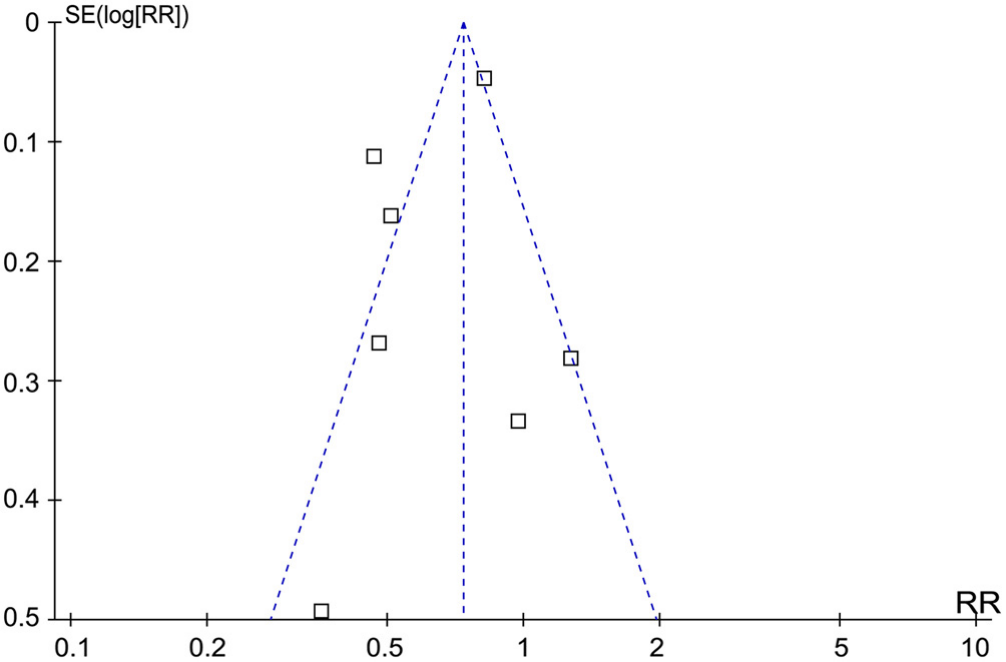
**Funnel plots for the meta-analysis of the influence of concurrent IABP on short-term mortality of ACS-related CS patients on ECMO.** IABP: Intra-aortic balloon pump; ACS: Acute coronary syndrome; CS: Cardiogenic shock; ECMO: Extracorporeal membrane oxygenation; SE: Standard error; RR: Risk ratio.

Funnel plots for the meta-analysis on the influence of concurrent IABP on short-term mortality of ACS-related CS patients treated with ECMO appeared symmetrical, suggesting a low likelihood of publication bias (Figure 6). This was further supported by Egger’s regression test (*P* ═ 0.37), indicating a low risk of publication bias.

## Discussion

This meta-analysis demonstrates that the concurrent use of IABP and VA-ECMO is associated with a significant reduction in short-term mortality in patients with ACS-related CS. By synthesizing data from seven cohort studies involving 5467 patients, we found that concurrent IABP and ECMO reduced mortality risk by 36% compared to ECMO alone. These findings provide further evidence supporting the combined use of IABP and ECMO in this high-risk population, and clinical trials are highly suggested to confirm these results. Several prior meta-analyses have suggested that concurrent IABP and ECMO may be superior to ECMO alone in reducing the short-term mortality of patients with AMI-related CS [13–15]. However, these earlier studies relied solely on crude mortality data. Given the absence of prospective randomized data, it is essential to perform meta-analyses using multivariate analyses to minimize the influence of potential confounding factors between groups. A major strength of the current meta-analysis is the inclusion of only multivariate studies, which enhances the robustness of the findings by minimizing confounding factors [30]. Nevertheless, it is important to acknowledge that all studies included in this meta-analysis are observational, which inherently introduces potential confounding factors that could influence the results [30]. Key confounders identified in previous studies include patient demographics, such as age [31] and sex [32], comorbid conditions like diabetes mellitus [33], and differences in ACS subtypes [34], all of which can impact mortality outcomes in patients with ACS-related CS. Additionally, renal function [35] and variability in intervention protocols, including the timing and duration of IABP and VA-ECMO use, are significant factors that might affect outcomes [36]. The timing of intervention relative to the onset of CS also plays a crucial role, as earlier intervention typically correlates with better outcomes [36]. Although our meta-analysis includes studies with multivariate adjustments to account for these factors, residual confounding may still exist due to the observational nature of the included studies. Accordingly, RCTs are needed to further validate these findings. The synergistic effects of IABP and ECMO can be attributed to their complementary mechanisms. IABP works by inflating during diastole and deflating during systole, which improves coronary perfusion by increasing diastolic pressure and reduces left ventricular afterload [37]. This reduction in afterload decreases myocardial oxygen demand and ventricular workload, which can be particularly beneficial in the context of CS, where the balance between myocardial oxygen supply and demand is critically compromised [37, 38]. VA-ECMO provides robust hemodynamic support by maintaining systemic circulation and oxygenation, effectively reducing the workload of the heart and allowing time for myocardial recovery [37, 38]. Together, these devices may optimize cardiac unloading and perfusion, reducing ischemia and promoting recovery of myocardial function. Meta-regression and subgroup analyses revealed that the proportion of men in each study significantly influenced the outcomes, with higher proportions of men correlating with more pronounced benefits of concurrent IABP and ECMO. However, we were unable to directly compare male and female patients due to the lack of individual-patient data in the included studies. Although these results should be interpreted with caution, as they are based on the proportion of men at the study level, this finding suggests that sex-specific physiological differences may impact the effectiveness of these interventions. Men and women differ in cardiovascular physiology and their response to mechanical support. For example, men typically have larger coronary artery diameters and different patterns of coronary artery disease compared to women, which could influence the hemodynamic effects of IABP [39]. Female patients with ACS-related CS tend to be older, have more severe comorbidities, and are more likely to present with non-AMI etiologies and preserved ejection fractions [40]. They are also more often treated with pharmacotherapy and less likely to receive mechanical circulatory support [41]. These factors may contribute to a higher mortality risk in female patients with ACS-related CS. These sex-specific differences warrant further investigation to understand their impact on the outcomes of combined IABP and ECMO therapy. Despite the strengths of our methodology, several limitations should be considered. As noted, all the included studies were observational, which, despite multivariate adjustments, cannot entirely eliminate the risk of residual confounding. Additionally, heterogeneity among studies was high, though much of it was explained by the proportion of men in each study. Another limitation is the lack of data on the long-term efficacy and safety of concurrent IABP and ECMO, as the included studies primarily reported short-term mortality. While the potential for publication bias was assessed as low, it cannot be entirely ruled out due to the limited number of available datasets [42]. Future research should aim to validate these findings through RCTs to definitively establish the benefits of concurrent IABP and ECMO in ACS-related CS. Such trials would provide higher-level evidence and could explore optimal patient selection criteria, timing, and duration of intervention. Additionally, further studies should investigate the underlying mechanisms and potential sex-specific differences in response to these combined interventions. Understanding the precise interactions between IABP and ECMO, and their impact on various patient subgroups, could lead to more personalized and effective treatment strategies. While our meta-analysis underscores the need for RCTs to establish causality and minimize bias, future RCTs should be meticulously designed to address the limitations identified in current observational studies. First, RCTs should incorporate stratification based on key confounding factors, such as age, sex, ACS subtype, and renal function, to ensure balanced allocation across treatment groups [43]. Additionally, clear criteria for defining and measuring “short-term” mortality should be established, standardizing the follow-up period (e.g., 30 days) to enable consistent comparisons. RCTs should also consider the timing and protocol of interventions, such as the initiation of IABP and VA-ECMO, to determine the optimal therapeutic window and reduce variability in outcomes [10, 44]. Moreover, collecting comprehensive data on comorbidities and concomitant treatments will allow for more robust multivariate analyses, helping to isolate the effects of the interventions from other influencing factors [45]. Finally, given the complexity of ACS-related CS, RCTs with larger sample sizes and multicenter collaboration will be essential to enhance the generalizability of findings and provide high-quality evidence to guide clinical practice.

## Conclusion

In conclusion, this meta-analysis provides further evidence that concurrent IABP and ECMO significantly reduce short-term mortality in patients with ACS-related CS. These findings support the use of combined mechanical support in this high-risk population and highlight the need for further research to optimize treatment protocols and validate these results through RCTs. Improved understanding of the mechanisms underlying these benefits and the influence of patient characteristics, such as sex, will be crucial in refining and enhancing the therapeutic approach for ACS-related CS.

## Supplemental data

**Table:** PubMed from inception to May 28, 2024

**#**	**Searches**	**Results**
**1**	“Extracorporeal membrane oxygenation”[All Fields] OR “ECMO”[All Fields] OR “heart-lung machine”[All Fields] OR “extracorporeal life support”[All Fields] OR “ECLS”[All Fields]	**30,067**
**2**	“intra-aortic balloon pump”[All Fields] OR “Intraaortic balloon pump”[All Fields] OR “IABP”[All Fields] OR “counterpulsation device”[All Fields]	**5432**
**3**	“shock”[All Fields] OR “cardiogenic shock”[All Fields] OR “cardiac shock”[All Fields]	**282,311**
**4**	“myocardial infarction”[All Fields] OR “acute coronary syndrome”[All Fields] OR “ACS”[All Fields] OR “AMI”[All Fields]	**479,826**
**5**	“combined”[All Fields] OR “concomitant”[All Fields] OR “concurrent”[All Fields] OR “simultaneous”[All Fields] OR “simultaneously”[All Fields] OR “random”[All Fields] OR “RCT”[All Fields] OR “randomized”[All Fields] OR “controlled”[All Fields] OR “matched”[All Fields] OR “adjusted”[All Fields] OR “adjustment”[All Fields] OR “adjusting”[All Fields] OR “logistic”[All Fields] OR “multivariate”[All Fields] OR “multivariable”[All Fields] OR “confounding”[All Fields] OR “propensity”[All Fields]	**585,3117**
**6**	1 and 2 and 3 and 4 and 5	**116**

**Table:** Web of Science from inception to May 28, 2024

**#**	**Searches**	**Results**
**1**	TS ═ “Extracorporeal membrane oxygenation” OR “ECMO” OR “heart-lung machine” OR “extracorporeal life support” OR “ECLS”	**31,092**
**2**	TS ═ “intra-aortic balloon pump” OR “Intraaortic balloon pump” OR “IABP” OR “counterpulsation device”	**5203**
**3**	TS ═ “shock” OR “cardiogenic shock” OR “cardiac shock”	**454,822**
**4**	TS ═ “myocardial infarction” OR “acute coronary syndrome” OR “ACS” OR “AMI”	**408,836**
**5**	TS ═ “combined” OR “concomitant” OR “concurrent” OR “simultaneous” OR “simultaneously” OR “random” OR “RCT” OR “randomized” OR “controlled” OR “matched” OR “adjusted” OR “adjustment” OR “adjusting” OR “logistic” OR “multivariate” OR “multivariable” OR “confounding” OR “propensity”	**902,0311**
**6**	1 and 2 and 3 and 4 and 5	**413**

**Table:** Embase from inception to May 28, 2024

**#**	**Searches**	**Results**
**1**	“extracorporeal membrane oxygenation” OR “ecmo” OR “heart-lung machine” OR “extracorporeal life support” OR “ecls”	**48,231**
**2**	“intra-aortic balloon pump” OR “intraaortic balloon pump” OR “iabp” OR “counterpulsation device”	**15,899**
**3**	“shock” OR “cardiogenic shock” OR “cardiac shock”	**449,522**
**4**	“myocardial infarction” OR “acute coronary syndrome” OR “acs” OR “ami”	**816,165**
**5**	“combined” OR “concomitant” OR “concurrent” OR “simultaneous” OR “simultaneously” OR “random” OR “rct” OR “randomized” OR “controlled” OR “matched” OR “adjusted” OR “adjustment” OR “adjusting” OR “logistic” OR “multivariate” OR “multivariable” OR “confounding” OR “propensity”	**1,4835,893**
**6**	1 and 2 and 3 and 4 and 5	**446**
**7**	Limit 6 to human	**418**
**8**	Limit 7 to clinical study	**288**
**9**	Limit 8 to Embase	**203**

## Data Availability

All the data generated during the study was within the manuscript.
